# Comparative analysis of adverse drug reaction reports for leukotriene receptor antagonists mainly indicated for allergic rhinitis and asthma: a WHO VigiAccess study

**DOI:** 10.3389/fmed.2026.1820058

**Published:** 2026-09-01

**Authors:** Youlin Dong, Zhigang Xia, Weimin Gao

**Affiliations:** Department of Otolaryngology, The Second Affiliated Hospital of Jiaxing University, Zhejiang, China

**Keywords:** adverse drug reactions, allergic rhinitis, disproportionality analysis, leukotriene receptor antagonists, neuropsychiatric ADRs, WHO VigiAccess

## Abstract

**Background:**

Leukotriene receptor antagonists (LTRAs), including montelukast, pranlukast, and zafirlukast, are generally well tolerated and widely used for asthma and allergic rhinitis, but they may cause adverse drug reactions (ADRs). We compared their real-world reporting profiles to identify potential agent-specific safety differences.

**Methods:**

We conducted a descriptive and disproportionality analysis of World Health Organization (WHO) VigiAccess data retrieved on August 1, 2025. Reports were included from each drug's first year available in VigiAccess (zafirlukast, 1997; montelukast, 1998; pranlukast, 2002) through August 1, 2025. Report-level demographics were summarized using the number of individual case safety reports (ICSRs), whereas System Organ Class (SOC)- and Preferred Term (PT)-level percentages used the total number of ADR entries for the corresponding drug as the denominator. Reporting odds ratios (RORs), proportional reporting ratios (PRRs), and 95% confidence intervals (CIs) compared each target LTRA with the other two LTRAs combined.

**Results:**

We identified 37,621 ICSRs: montelukast, 33,615 (89.4%); pranlukast, 2,109 (5.6%); and zafirlukast, 1,897 (5.0%). Among 96,624 montelukast ADR entries, the SOC Psychiatric disorders accounted for *n* = 29,498 (30.5%). Positive PT-level signals included nightmares (*n* = 1,688, 1.75%; ROR = 45.89, 95% CI: 14.78–142.45; PRR = 45.10, 95% CI: 14.53–139.95), aggression (*n* = 1,758, 1.82%; ROR = 28.69, 95% CI: 11.92–69.03; PRR = 28.18, 95% CI: 11.72–67.78), and suicidal ideation (*n* = 1,901, 1.97%; ROR = 19.41, 95% CI: 9.69–38.88; PRR = 19.05, 95% CI: 9.52–38.13). Among 2,724 pranlukast ADR entries, Gastrointestinal disorders accounted for *n* = 809 (29.7%). Zafirlukast showed disproportionate hepatobiliary reporting (ROR = 5.87, 95% CI: 4.88–7.06; PRR = 5.72, 95% CI: 4.78–6.85). Seven neuropsychiatric PTs were shared across all three agents; their counts, ADR-entry proportions, and RORs ranged from 569 to 2,313, 0.59%–2.39%, and 1.22–19.41 for montelukast; 2–99, 0.07%–3.63%, and 0.03–1.82 for pranlukast; and 4–35, 0.08%–0.70%, and 0.06–0.32 for zafirlukast.

**Conclusions:**

Real-world evidence from spontaneous-report databases suggests differences in the safety-reporting profiles of montelukast, pranlukast, and zafirlukast. However, under-reporting and incomplete clinical information preclude estimation of incidence or causal inference; these findings should be interpreted as safety signals.

## 1 Introduction

Asthma and allergic rhinitis are chronic inflammatory airway diseases that together affect hundreds of millions of people worldwide and impose a substantial global health and economic burden (1, 2). A recent network meta-analysis evaluating regular and as-needed therapies for mild asthma included leukotriene receptor antagonists (LTRAs) as controller regimens and found that regular inhaled corticosteroids provided better exacerbation prevention than LTRAs in children (3). Montelukast, pranlukast, and zafirlukast antagonize the cysteinyl leukotriene type-1 (CysLT_1_) receptor, thereby attenuating leukotriene-mediated bronchoconstriction, microvascular permeability, and mucus hypersecretion (4, 5). Montelukast is generally administered once daily, whereas zafirlukast and pranlukast generally require twice-daily administration.

Despite their established efficacy and broad use, LTRA safety requires continued evaluation. Clinical studies and post-marketing surveillance indicate that these agents are generally well tolerated, although neuropsychiatric and hepatic adverse drug reactions (ADRs) have been reported (6–8). Spontaneous-reporting systems such as WHO VigiAccess complement clinical evidence by supporting detection of rare, delayed, or previously unrecognized ADR patterns (9).

Comparative evidence on LTRA safety remains limited and has primarily addressed selected agents rather than all three principal LTRAs (10, 11). Existing studies have often focused on individual drugs or selected ADRs, leaving uncertainty about whether montelukast, pranlukast, and zafirlukast show different spontaneous-reporting patterns.

We therefore used VigiAccess data to compare the reported ADR profiles of montelukast, pranlukast, and zafirlukast. The objectives were to describe the spectrum and frequency of reported ADRs for each agent and to identify drug–ADR combinations with disproportionate reporting.

## 2 Methods

### 2.1 Search strategy and data source

The World Health Organization (WHO) VigiAccess database (https://www.vigiaccess.org), which provides public access to aggregated data from VigiBase, the WHO global database of individual case safety reports (ICSRs), was used as the data source for this study (12). Reports listing montelukast, pranlukast, or zafirlukast as suspected or interacting medicinal products were retrieved on August 1, 2025. The inclusion period began with the first report year displayed for each drug in VigiAccess—1997 for zafirlukast, 1998 for montelukast, and 2002 for pranlukast—and ended on August 1, 2025.

For each ICSR, we obtained the aggregated variables provided by VigiAccess: patient age group, sex, calendar year of reporting, geographical region (Africa, Americas, Asia, Europe, and Oceania), and reported ADRs. ADRs are coded using the Medical Dictionary for Regulatory Activities (MedDRA). A System Organ Class (SOC) is the highest-level grouping of related medical concepts, whereas a Preferred Term (PT) represents a distinct clinical concept used to describe an ADR (13). The analysis included 27 SOC categories, including non-clinical groupings such as Product issues (product quality, supply, packaging, or use) and Social circumstances (relevant social or lifestyle circumstances). Report-level percentages used the number of ICSRs for the corresponding drug as the denominator. SOC- and PT-level frequencies represented ADR entries, not patients or reports. Because one ICSR may contain multiple ADRs, percentages used all ADR entries for each drug (montelukast, *N* = 96,624; pranlukast, *N* = 2,724; zafirlukast, *N* = 5,021).

Because VigiAccess provides anonymized, aggregated pharmacovigilance data derived from spontaneous reporting systems in more than 150 countries participating in the WHO Programme for International Drug Monitoring, no individual patient identifiers are available and ethics committee approval was not required.

### 2.2 Disproportionality analysis

We performed disproportionality analyses using the reporting odds ratio (ROR) and proportional reporting ratio (PRR) (14, 15). For each drug–ADR pair, a 2 × 2 table was constructed: a, reports of the specified ADR for the target LTRA; b, reports of all other ADRs for the target LTRA; c, reports of the specified ADR for the other two LTRAs combined; and d, reports of all other ADRs for the other two LTRAs combined. The ROR compares reporting odds, whereas the PRR compares reporting proportions.

The ROR and PRR were then calculated as:

$$\mathrm{ROR}=\frac{a/c}{b/d}$$

$$\mathrm{PRR}=\frac{a/(a+b)}{c/(c+d)}$$

ROR was calculated as ad/bc and PRR as [*a*/(*a* + *b*)]/[*c*/(*c* + *d*)]. A potential signal required at least three reports (*a* ≥ 3), ROR > 2.0, PRR > 2.0, and lower 95% CI bounds > 1.0 for both measures. Signals were retained only when the ROR- and PRR-based criteria were concordantly positive.

### 2.3 Statistical analysis

Descriptive statistics were used to summarize the characteristics of ADR reports for each LTRA. Sex, age group, geographical region, reporting year, SOC, and PT were summarized as counts and percentages. Data extraction and tabulation were performed using Microsoft Excel. All SOC- and PT-level RORs, PRRs, and corresponding 95% confidence intervals (CIs) were calculated using R version 4.5.2 according to the prespecified formulas. Signal status was determined using the joint criteria described above. Heatmaps displayed the magnitude of ROR and PRR across SOCs and selected PTs, facilitating comparison of LTRA-specific ADR reporting patterns.

## 3 Results

### 3.1 Case description of the study

This study analyzed pharmacovigilance reports for montelukast, pranlukast, and zafirlukast (Table 1). The earliest reports were recorded in 1997 for zafirlukast, 1998 for montelukast, and 2002 for pranlukast. As of August 1, 2025, 33,615 reports were identified for montelukast (89.4%), 2,109 for pranlukast (5.6%), and 1,897 for zafirlukast (5.0%). Females accounted for 55.3%, 58.2%, and 62.9% of reports, respectively. The largest reported age category was <18 years for montelukast (28.6%) and 45–64 years for pranlukast (31.2%) and zafirlukast (32.7%). The Americas contributed 45.1% of montelukast and 79.5% of zafirlukast reports, whereas Asia contributed 98.9% of pranlukast reports.

**Table 1:** Demographic characteristics of adverse drug reaction reports for montelukast, pranlukast, and zafirlukast.

Demographic characteristic	Montelukast *N* = 33,615 ICSRs	Pranlukast *N* = 2,109 ICSRs	Zafirlukast *N* = 1,897 ICSRs
First report year	1998	2002	1997
Number of ADR reports	33,615 (89.4%)	2,109 (5.6%)	1,897 (5.0%)
Sex, *n* (%)			
Female	18,589 (55.3%)	1,227 (58.2%)	1,194 (62.9%)
Male	12,870 (38.3%)	808 (38.3%)	531 (28.0%)
Unknown	2,156 (6.4%)	74 (3.5%)	172 (9.1%)
Age, years, *n* (%)			
<18	9,616 (28.6%)	392 (18.6%)	81 (4.3%)
18–44	5,853 (17.4%)	330 (15.6%)	380 (20.0%)
45–64	6,601 (19.6%)	658 (31.2%)	620 (32.7%)
65–74	2,809 (8.4%)	431 (20.4%)	259 (13.7%)
>75	1,705 (5.1%)	240 (11.4%)	102 (5.4%)
Unknown	7,031 (20.9%)	58 (2.8%)	455 (24.0%)
Region, *n* (%)			
Africa	372 (1.1%)	0 (0.0%)	3 (0.2%)
Americas	15,161 (45.1%)	24 (1.1%)	1,509 (79.5%)
Asia	7,784 (23.2%)	2,085 (98.9%)	47 (2.5%)
Europe	9,583 (28.5%)	0 (0.0%)	332 (17.5%)
Oceania	715 (2.1%)	0 (0.0%)	6 (0.3%)
Report year, *n* (%)			
2025	1,075 (3.2%)	133 (6.3%)	6 (0.3%)
2024	2,094 (6.2%)	305 (14.5%)	9 (0.5%)
2023	1,891 (5.6%)	251 (11.9%)	3 (0.2%)
2022	1,704 (5.1%)	3 (0.1%)	5 (0.3%)
2021	1,473 (4.4%)	102 (4.8%)	2 (0.1%)
2020	2,104 (6.3%)	366 (17.4%)	3 (0.2%)
2019	2,565 (7.6%)	154 (7.3%)	13 (0.7%)
Before 2019	20,709 (61.6%)	795 (37.7%)	1,856 (97.8%)

Values are *n* (%) unless otherwise indicated. ADR, adverse drug reaction; ICSR, individual case safety report. Percentages for sex, age, region, and report year use the drug-specific ICSR total; percentages in “Number of ADR reports” use all 37,621 ICSRs.

### 3.2 Distribution tables of 27 SOCs for three leukotriene receptor antagonists

Table 2 summarizes 96,624 montelukast, 2,724 pranlukast, and 5,021 zafirlukast ADR entries across 27 SOCs. Psychiatric disorders accounted for *n* = 29,498 (30.5%) of montelukast entries, *n* = 152 (5.6%) of pranlukast entries, and *n* = 225 (4.5%) of zafirlukast entries. Gastrointestinal disorders were most prominent for pranlukast (*n* = 809, 29.7%), compared with montelukast (*n* = 7,783, 8.1%) and zafirlukast (*n* = 417, 8.3%). For zafirlukast, Respiratory, thoracic and mediastinal disorders accounted for *n* = 581 (11.6%), and General disorders and administration site conditions for *n* = 710 (14.1%).

**Table 2:** Distribution of adverse drug reaction entries across 27 system organ classes for montelukast, pranlukast, and zafirlukast.

System organ class	Montelukast *N* = 96,624 ADR	Pranlukast *N* = 2,724 ADR	Zafirlukast *N* = 5,021 ADR
Blood and lymphatic system	684 (0.7%)	15 (0.6%)	131 (2.6%)
Cardiac	1,465 (1.5%)	48 (1.8%)	164 (3.3%)
Congenital, familial and genetic	176 (0.2%)	1 (<0.1%)	4 (0.1%)
Ear and labyrinth	480 (0.5%)	6 (0.2%)	16 (0.3%)
Endocrine	125 (0.1%)	1 (<0.1%)	14 (0.3%)
Eye	1,134 (1.2%)	25 (0.9%)	62 (1.2%)
Gastrointestinal	7,783 (8.1%)	809 (29.7%)	417 (8.3%)
General disorders and administration site conditions	11,147 (11.5%)	189 (6.9%)	710 (14.1%)
Hepatobiliary	493 (0.5%)	22 (0.8%)	149 (3.0%)
Immune system	1,891 (2.0%)	40 (1.5%)	161 (3.2%)
Infections and infestations	2,333 (2.4%)	86 (3.2%)	193 (3.8%)
Injury, poisoning and procedural complications	4,111 (4.2%)	152 (5.6%)	258 (5.1%)
Investigations	2,711 (2.8%)	55 (2.0%)	303 (6.0%)
Metabolism and nutrition	961 (1.0%)	41 (1.5%)	88 (1.8%)
Musculoskeletal and connective tissue disorders	3,296 (3.4%)	43 (1.6%)	303 (6.0%)
Neoplasms benign, malignant and unspecified (incl cysts and polyps)	168 (0.2%)	3 (0.1%)	31 (0.6%)
Nervous system	10,236 (10.6%)	544 (20.0%)	490 (9.8%)
Pregnancy, puerperium and perinatal conditions	354 (0.4%)	2 (0.1%)	7 (0.1%)
Psychiatric	29,498 (30.5%)	152 (5.6%)	225 (4.5%)
Renal and urinary	692 (0.7%)	59 (2.2%)	63 (1.2%)
Reproductive system and breast disorders	364 (0.4%)	6 (0.2%)	36 (0.7%)
Respiratory, thoracic and mediastinal disorders	7,641 (7.9%)	118 (4.3%)	581 (11.6%)
Skin and subcutaneous tissue disorders	5,967 (6.2%)	277 (10.2%)	431 (8.6%)
Social circumstances	533 (0.6%)	0 (0.0%)	10 (0.2%)
Surgical and medical procedures	436 (0.5%)	1 (<0.1%)	10 (0.2%)
Vascular	957 (1.0%)	29 (1.1%)	134 (2.7%)
Product issues	988 (1.0%)	0 (0.0%)	30 (0.6%)

Values are *n* (%). ADR, adverse drug reaction; SOC, System Organ Class. Percentages use the total ADR for montelukast (*N* = 96,624), pranlukast (*N* = 2,724), and zafirlukast (*N* = 5,021). Abbreviated labels are used for presentation; analyses used the full official MedDRA SOC names.

### 3.3 Top 20 reported ADRs for the three leukotriene receptor antagonists

The top 20 reported ADRs for each LTRA are shown in Table 3. Percentages represent proportions of all ADR entries for the corresponding drug. Montelukast entries most frequently included depression (2.4%), anxiety (2.4%), insomnia (2.1%), and headache (2.1%). Pranlukast entries most frequently included somnolence (10.9%), dry mouth (8.0%), diarrhoea (5.5%), and dyspepsia (4.4%). Zafirlukast entries most frequently included dyspnoea (2.3%), asthma (2.3%), headache (2.2%), and rash (1.8%).

**Table 3:** Top 20 reported adverse drug reaction preferred terms for montelukast, pranlukast, and zafirlukast.

Montelukast		Pranlukast		Zafirlukast	
Preferred term	*n* (%)	Preferred term	*n* (%)	Preferred term	*n* (%)
Depression	2,313 (2.4%)	Somnolence	296 (10.9%)	Dyspnoea	116 (2.3%)
Anxiety	2,307 (2.4%)	Dry mouth	217 (8.0%)	Asthma	115 (2.3%)
Insomnia	2,026 (2.1%)	Diarrhoea	150 (5.5%)	Headache	109 (2.2%)
Headache	2,010 (2.1%)	Dyspepsia	121 (4.4%)	Rash	92 (1.8%)
Suicidal ideation	1,901 (2.0%)	Insomnia	99 (3.6%)	Eosinophilic granulomatosis with polyangiitis	81 (1.6%)
Aggression	1,758 (1.8%)	Dizziness	90 (3.3%)	Malaise	75 (1.5%)
Drug ineffective	1,705 (1.8%)	Product prescribing error	85 (3.1%)	Nausea	69 (1.4%)
Nightmare	1,688 (1.8%)	Pruritus	83 (3.0%)	Pruritus	68 (1.4%)
Dyspnoea	1,271 (1.3%)	Constipation	79 (2.9%)	Arthralgia	63 (1.2%)
Asthma	1,175 (1.2%)	Rash	66 (2.4%)	Bronchospasm	63 (1.2%)
Rash	1,150 (1.2%)	Nausea	55 (2.0%)	Eosinophilia	62 (1.2%)
Pruritus	1,123 (1.2%)	Urticaria	53 (1.9%)	Product dose omission issue	62 (1.2%)
Dizziness	1,103 (1.1%)	Intercepted medication error	47 (1.7%)	Abdominal pain	61 (1.2%)
Abnormal behaviour	1,068 (1.1%)	Pyrexia	45 (1.6%)	Fatigue	61 (1.2%)
Diarrhoea	1,059 (1.1%)	Abdominal pain	44 (1.6%)	Condition aggravated	56 (1.1%)
Nausea	1,039 (1.1%)	Headache	44 (1.6%)	Chest pain	54 (1.1%)
Somnolence	1,007 (1.0%)	Vomiting	43 (1.6%)	Myalgia	54 (1.1%)
Fatigue	993 (1.0%)	Face oedema	32 (1.2%)	Urticaria	49 (1.0%)
Cough	932 (1.0%)	Palpitations	32 (1.2%)	Vasculitis	49 (1.0%)
Irritability	929 (1.0%)	Abdominal discomfort	30 (1.1%)	Dizziness	43 (0.9%)

Values are *n* (%). ADR, adverse drug reaction; ICSR, individual case safety report; PT, Preferred Term. Percentages use all ADR for the corresponding drug as the denominator: montelukast, *N* = 96,624; pranlukast, *N* = 2,724; zafirlukast, *N* = 5,021.

### 3.4 Disproportionality analysis

**Figure 1:**
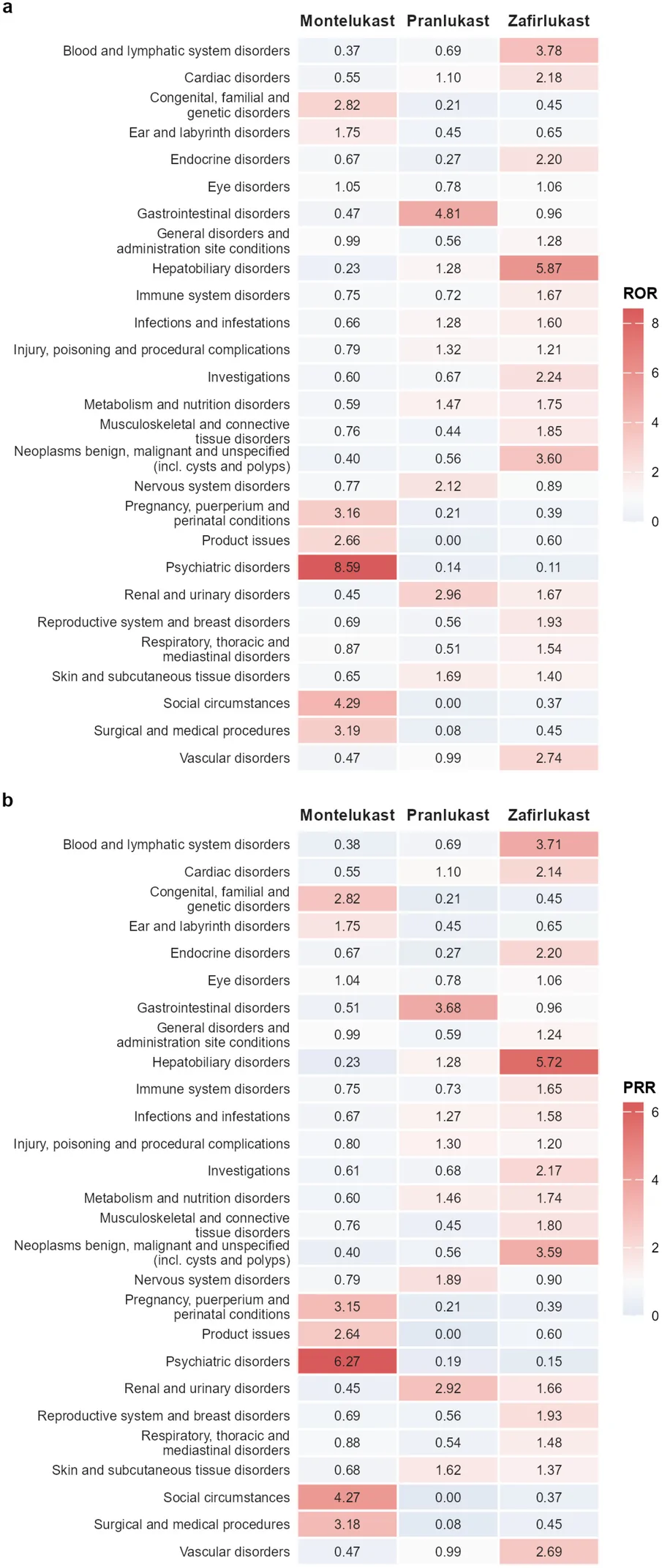
Disproportionality profiles across system organ classes. **(a)** Reporting odds ratios. **(b)** Proportional reporting ratios. Comparators were the other two leukotriene receptor antagonists combined.

Figure 1 shows ROR and PRR point estimates across SOCs; positive signals meeting all prespecified criteria are reported with 95% CIs in Table 4. Montelukast showed positive signals for psychiatric disorders, social circumstances, surgical and medical procedures, pregnancy/puerperium/perinatal conditions, congenital/familial/genetic disorders, and product issues. Pranlukast showed positive signals for gastrointestinal and renal/urinary disorders. Zafirlukast showed positive signals for blood/lymphatic, cardiac, endocrine, hepatobiliary, investigations, neoplasms, and vascular SOCs.

**Table 4:** Positive system organ class-level disproportionality signals meeting all prespecified criteria.

Drug	System organ class	ROR (95% CI)	PRR (95% CI)
Montelukast	Congenital, familial and genetic disorders	2.82 (1.16–6.87)	2.82 (1.16–6.86)
Montelukast	Pregnancy, puerperium and perinatal conditions	3.16 (1.63–6.13)	3.15 (1.63–6.11)
	Product issues	2.66 (1.85–3.82)	2.64 (1.84–3.79)
	Psychiatric disorders	8.59 (7.74–9.53)	6.27 (5.68–6.92)
	Social circumstances	4.29 (2.29–8.02)	4.27 (2.29–7.98)
	Surgical and medical procedures	3.19 (1.75–5.80)	3.18 (1.75–5.78)
Pranlukast	Gastrointestinal disorders	4.81 (4.42–5.24)	3.68 (3.46–3.91)
	Renal and urinary disorders	2.96 (2.26–3.87)	2.92 (2.24–3.79)
Zafirlukast	Blood and lymphatic system disorders	3.78 (3.13–4.57)	3.71 (3.08–4.46)
	Cardiac disorders	2.18 (1.85–2.57)	2.14 (1.83–2.51)
	Endocrine disorders	2.20 (1.27–3.83)	2.20 (1.27–3.82)
	Hepatobiliary disorders	5.87 (4.88–7.06)	5.72 (4.78–6.85)
	Investigations	2.24 (1.98–2.53)	2.17 (1.93–2.43)
	Neoplasms benign, malignant and unspecified (incl cysts and polyps)	3.60 (2.46–5.29)	3.59 (2.45–5.25)
	Vascular disorders	2.74 (2.28–3.28)	2.69 (2.25–3.21)

CI, confidence interval; PRR, proportional reporting ratio; ROR, reporting odds ratio; SOC, System Organ Class.

### 3.5 Top 20 neuropsychiatric ADRs of montelukast

PT-level analysis identified strong disproportionate reporting of several neuropsychiatric ADRs for montelukast (Table 5). Nightmares had ROR = 45.89 (95% CI: 14.78–142.45) and PRR = 45.10 (95% CI: 14.53–139.95), followed by aggression (ROR = 28.69, 95% CI: 11.92–69.03; PRR = 28.18, 95% CI: 11.72–67.78) and suicidal ideation (ROR = 19.41, 95% CI: 9.69–38.88; PRR = 19.05, 95% CI: 9.52–38.13).

**Table 5:** Top 20 neuropsychiatric adverse drug reactions reported for montelukast, with disproportionality estimates and 95% confidence intervals.

Montelukast			
ADR	Number	ROR (95% CI)	PRR (95% CI)
Nightmare	1,688	45.89 (14.78–142.45)	45.10 (14.53–139.95)
Aggression	1,758	28.69 (11.92–69.03)	28.18 (11.72–67.78)
Suicidal ideation	1,901	19.41 (9.69–38.88)	19.05 (9.52–38.13)
Abnormal behaviour	1,068	17.30 (7.18–41.67)	17.12 (7.11–41.21)
Hallucination	713	9.59 (4.29–21.42)	9.53 (4.27–21.27)
Abnormal dreams	454	9.14 (3.41–24.45)	9.10 (3.40–24.34)
Irritability	929	8.34 (4.33–16.09)	8.27 (4.29–15.95)
Fear	305	8.17 (2.62–25.48)	8.15 (2.61–25.40)
Anxiety	2,307	6.74 (4.64–9.79)	6.60 (4.55–9.58)
Agitation	835	6.13 (3.38–11.12)	6.08 (3.36–11.02)
Sleep disorder	817	6.00 (3.31–10.87)	5.95 (3.29–10.79)
Completed suicide	214	5.73 (1.83–17.91)	5.72 (1.83–17.87)
Thinking abnormal	203	5.43 (1.74–16.99)	5.42 (1.74–16.95)
Depression	2,313	5.40 (3.87–7.55)	5.30 (3.80–7.39)
Mood altered	569	5.09 (2.63–9.84)	5.07 (2.62–9.79)
Emotional disorder	292	4.69 (1.94–11.36)	4.68 (1.93–11.33)
Middle insomnia	149	3.99 (1.27–12.50)	3.98 (1.27–12.48)
Attention deficit hyperactivity disorder	214	3.44 (1.42–8.34)	3.43 (1.41–8.32)
Stress	124	2.49 (0.92–6.73)	2.48 (0.92–6.72)
Nervousness	278	2.48 (1.28–4.82)	2.48 (1.28–4.81)

ADR, adverse drug reaction; CI, confidence interval; PRR, proportional reporting ratio; ROR, reporting odds ratio.

Other positive montelukast PT-level signals included abnormal behaviour, hallucination, abnormal dreams, irritability, fear, anxiety, agitation, sleep disorder, completed suicide, thinking abnormal, depression, mood altered, emotional disorder, middle insomnia, attention deficit hyperactivity disorder, and nervousness. Full estimates are provided in Table 5.

### 3.6 Common and distinct neuropsychiatric ADRs of three leukotriene receptor antagonists

Seven neuropsychiatric PTs were reported for all three LTRAs: suicidal ideation, anxiety, agitation, mood altered, hallucination, depression, and insomnia (Table 6). Counts and percentages in Table 6 refer to ADR entries and the drug-specific ADR-entry denominator, respectively; their RORs differed by agent, with higher RORs for six PTs in montelukast entries.

**Table 6:** Shared and agent-specific neuropsychiatric preferred terms reported for the three leukotriene receptor antagonists.

Shared PT	Montelukast *N* = 96,624 ADR entries	Pranlukast *N* = 2,724 ADR entries	Zafirlukast *N* = 5,021 ADR entries
Suicidal ideation	*n* = 1,901 (1.97%); ROR = 19.41 (9.69–38.88)	*n* = 2 (0.07%); ROR = 0.04 (0.01–0.15)	*n* = 6 (0.12%); ROR = 0.06 (0.03–0.14)
Anxiety	*n* = 2,307 (2.39%); ROR = 6.74 (4.64–9.79)	*n* = 3 (0.11%); ROR = 0.05 (0.02–0.15)	*n* = 25 (0.50%); ROR = 0.21 (0.14–0.31)
Agitation	*n* = 835 (0.86%); ROR = 6.13 (3.38–11.12)	*n* = 5 (0.18%); ROR = 0.22 (0.09–0.53)	*n* = 6 (0.12%); ROR = 0.14 (0.06–0.31)
Mood altered	*n* = 569 (0.59%); ROR = 5.09 (2.63–9.84)	*n* = 4 (0.15%); ROR = 0.26 (0.10–0.69)	*n* = 5 (0.10%); ROR = 0.17 (0.07–0.41)
Hallucination	*n* = 713 (0.74%); ROR = 9.59 (4.29–21.42)	*n* = 2 (0.07%); ROR = 0.10 (0.03–0.41)	*n* = 4 (0.08%); ROR = 0.11 (0.04–0.29)
Depression	*n* = 2,313 (2.39%); ROR = 5.40 (3.87–7.55)	*n* = 2 (0.07%); ROR = 0.03 (0.01–0.12)	*n* = 33 (0.66%); ROR = 0.28 (0.20–0.39)
Insomnia	*n* = 2,026 (2.10%); ROR = 1.22 (1.02–1.45)	*n* = 99 (3.63%); ROR = 1.82 (1.48–2.24)	*n* = 35 (0.70%); ROR = 0.32 (0.23–0.45)
Agent-specific PTs	Mental disorder; neuropsychological symptoms; suicide attempt; personality change; restlessness; tic; anger; depressed mood; panic attack; fear; sleep terror; mood swings	Listlessness; delirium	Thinking abnormal; affective lability; personality disorder; stress

ADR, adverse drug reaction; CI, confidence interval; PT, Preferred Term; ROR, reporting odds ratio.

## 4 Discussion

VigiAccess supports post-marketing characterization of ADR reporting patterns that may not be fully captured in clinical trials (9). In this study, montelukast, pranlukast, and zafirlukast showed distinct and overlapping disproportionality profiles. We used disproportionality analysis to identify potential reporting signals and characterize differences in reporting patterns among these LTRAs.

Regional differences in report counts may reflect authorization and market duration, prescribing volume, and national pharmacovigilance organization (16, 17). Specific determinants include mandatory vs. voluntary reporting, reporter eligibility, electronic reporting access, reporter training, regulatory safety communications, and the capacity to receive, code, deduplicate, assess, and transmit reports to VigiBase. The concentration of pranlukast reports in Asia and zafirlukast reports in the Americas may therefore reflect regional availability and reporting pathways rather than drug-related incidence.

Montelukast showed the most pronounced neuropsychiatric reporting pattern, including depression, anxiety, insomnia, suicidal ideation, nightmares, aggression, abnormal behaviour, and hallucinations, consistent with regulatory, clinical, and systematic evidence (18, 19). Biological plausibility is supported by cysteinyl leukotriene receptors in brain regions involved in emotion, sleep, memory, and behaviour (20), and by experimental links between leukotriene signalling, neuroinflammation, microglial activation, neuronal injury, and blood–brain barrier disruption. Montelukast and pranlukast have also produced neuroprotective effects in experimental ischaemia and ageing models (21, 22), indicating that central responses may vary with dose, developmental stage, disease context, and downstream signalling. A human forebrain-organoid study further implicated adenylate cyclase 1 (ADCY1), reduced cyclic adenosine monophosphate (cAMP) signalling, altered neuronal gene expression, impaired maturation, and reduced neural activity (23). Together, these findings support biological plausibility but do not establish causality. Signals involving pregnancy/perinatal conditions, social circumstances, congenital/genetic disorders, product issues, and medical procedures require replication and case-level assessment.

Pranlukast showed a distinct pattern dominated by gastrointestinal entries, with somnolence, insomnia, dizziness, and other nervous-system or sleep-related events also appearing descriptively. Korean pharmacovigilance data similarly identified gastrointestinal and neuropsychiatric reports (10), and a randomized crossover study reported abdominal pain, diarrhoea, and liver-enzyme or bilirubin elevations (24). Oral absorption, high protein binding, variable exposure, and a limited gastrointestinal absorption window provide pharmacological context but do not explain the observed predominance (25, 26). The less-characterized renal/urinary signal requires case-level assessment of renal function, hydration, nephrotoxic co-medication, timing, and dechallenge/rechallenge.

Zafirlukast showed prominent respiratory and systemic entries and the strongest hepatobiliary disproportionality signal. Its extensive hepatic metabolism, principally through cytochrome P450 2C9 (CYP2C9), and *in vitro* inhibition of CYP2C9 and cytochrome P450 3A (CYP3A) are consistent with increased warfarin effects and liver-enzyme elevations (8). Eosinophilic granulomatosis with polyangiitis has also been reported during LTRA treatment, including zafirlukast, particularly during systemic corticosteroid tapering (27). Nathani et al. proposed that this temporal pattern may reflect unmasking of previously suppressed eosinophilic vasculitis (27). Assessment should therefore consider corticosteroid exposure, eosinophilia, systemic symptoms, and temporal sequence.

Seven neuropsychiatric PTs were shared across the three LTRAs: suicidal ideation, anxiety, agitation, altered mood, hallucinations, depression, and insomnia. Their ADR-entry proportions and RORs differed markedly, with the broadest pattern for montelukast. Comparative evidence likewise documents neuropsychiatric concerns most consistently for montelukast, while evidence for pranlukast and zafirlukast is more limited (11, 19). The shared PTs may suggest a class component, but differences in exposure, patient mix, indications, regional use, regulatory attention, and reporting behaviour may also affect signal magnitude. Clinicians should remain alert to new sleep, mood, or behavioural symptoms during LTRA treatment, particularly in children and patients with pre-existing neuropsychiatric conditions.

This study has several limitations. Spontaneous reporting systems are affected by substantial under-reporting, selective reporting, missing data, duplicate reports, and the absence of exposure denominators (28). Notoriety bias following regulatory warnings or publicity may alter reporting frequency and disproportionality estimates (29–32). Reports frequently lacked previous medical history, concomitant diseases and therapies, treatment duration, and dechallenge/rechallenge information, limiting causal assessment and adjustment for confounding. Differences in approval dates, geographic availability, prescribing, and reporting practices further complicate direct comparisons. Zileuton was excluded because the prespecified comparison concerned cysteinyl leukotriene receptor antagonists; its distinct mechanism warrants separate evaluation.

## 5 Conclusion

Although LTRAs are generally well tolerated and considered safe, they may cause ADRs. Real-world evidence from spontaneous-report databases suggests differences in the safety-reporting profiles of montelukast, pranlukast, and zafirlukast. However, under-reporting prevents estimation of incidence or absolute risk, and limited information on medical history, comorbidities, treatment duration, dechallenge/rechallenge, and concomitant therapies constrains causal assessment and control of confounding. The observed disproportionality findings should therefore be interpreted as safety signals requiring further investigation rather than evidence of causal associations.

## Data Availability

Publicly available datasets were analyzed in this study. This data can be found here: Publicly available datasets were analyzed in this study. This data can be found here: https://www.vigiaccess.org.
